# Complete Genome Sequence of Stenotrophomonas maltophilia Siphophage Suzuki

**DOI:** 10.1128/mra.00136-22

**Published:** 2022-03-08

**Authors:** Teresa Sullivan, Nikhil Manuel, James Clark, Mei Liu, Ben Burrowes

**Affiliations:** a Department of Biochemistry and Biophysics, Texas A&M University, College Station, Texas, USA; b Center for Phage Technology, Texas A&M University, College Station, Texas, USA; c BB Phage Consultancy, LLC, Georgetown, Texas, USA; Queens College CUNY

## Abstract

Stenotrophomonas maltophilia is a Gram-negative bacterium known to cause respiratory tract infections and other diseases in humans. Here, we describe the isolation and genome annotation of S. maltophilia siphophage Suzuki. Its 56,042-bp genome has 83 predicted protein-coding genes and demonstrates similarity with *Xylella* phages Sano and Salvo.

## ANNOUNCEMENT

Stenotrophomonas maltophilia is a Gram-negative bacterium found in the environment, most commonly aquatic habitats. Since it is a multidrug-resistant pathogen capable of causing human infection, it is especially concerning for immunocompromised individuals (1). Due to the emergence of multidrug-resistant organisms, phage therapy could play an important role by offering an alternative treatment modality to traditional therapeutic antibacterials (2). Here, we present the complete annotated genome sequence of S. maltophilia siphophage Suzuki.

Phage Suzuki was isolated as previously described (3) from a freshwater sample collected from Bastrop Bayou in Richwood, TX (29.147505, -95.314471), in 2019. Suzuki was isolated using S. maltophilia (ATCC 17807) as propagation host using the soft agar overlay method described by Adams (3). Host bacteria were cultured aerobically at 30°C in tryptone nutrient broth (0.5% tryptone, 0.25% yeast extract, 0.1% glucose, and 0.85% NaCl [wt/vol]). Phage genomic DNA was purified using a Promega Wizard DNA cleanup system as previously described (4). DNA sequencing libraries were prepared as 300-bp inserts using a Swift 2S Turbo kit. and the prepared libraries were sequenced on an Illumina MiSeq with paired-end 150-bp reads using v2 300-cycle chemistry. Raw reads were quality controlled using FastQC (https://www.bioinformatics.babraham.ac.uk/projects/fastqc/) and trimmed with FastX-Toolkit v0.11.6 (http://hannonlab.cshl.edu/fastx_toolkit/), and the resulting 72,362 trimmed reads were assembled using SPAdes v3.5.0 (5) into a single contig at 57-fold coverage. The complete sequence of the contig assembly was confirmed by Sanger sequencing of a PCR product amplified off the contig ends (primer sequences, 5′-CAGTGAACACGCCTGCATC-3′ and 5′-ACTCGCAGTAGCAAATCGCA-3′). The completed genome sequence was annotated using the CPT Galaxy-Apollo phage annotation platform (https://cpt.tamu.edu/galaxy-pub) (6–8). Structural annotations were performed using Glimmer v3 and MetaGeneAnnotator v1.0, and tRNA sequences were detected using ARAGORN v2.36 and tRNAScan-SE v2.0 (9–12). Gene functions were predicted using InterProScan v5.48, BLAST v2.9.0, TMHMM v2.0, HHPred, LipoP v1.0, and SignalP v5.0 (13–18). BLAST searches were executed against the NCBI nonredundant and UniProtKB Swiss-Prot/TrEMBL databases with a 0.001 maximum expectation value cutoff (19). Genome-wide DNA sequence similarity was calculated by progressiveMauve v2.4 (20). All tools were run with default settings unless otherwise specified. Phage morphology was determined by negatively staining with 2% (wt/vol) uranyl acetate and viewing by transmission electron microscopy at the Texas A&M Microscopy and Imaging Center.

**FIG 1:**
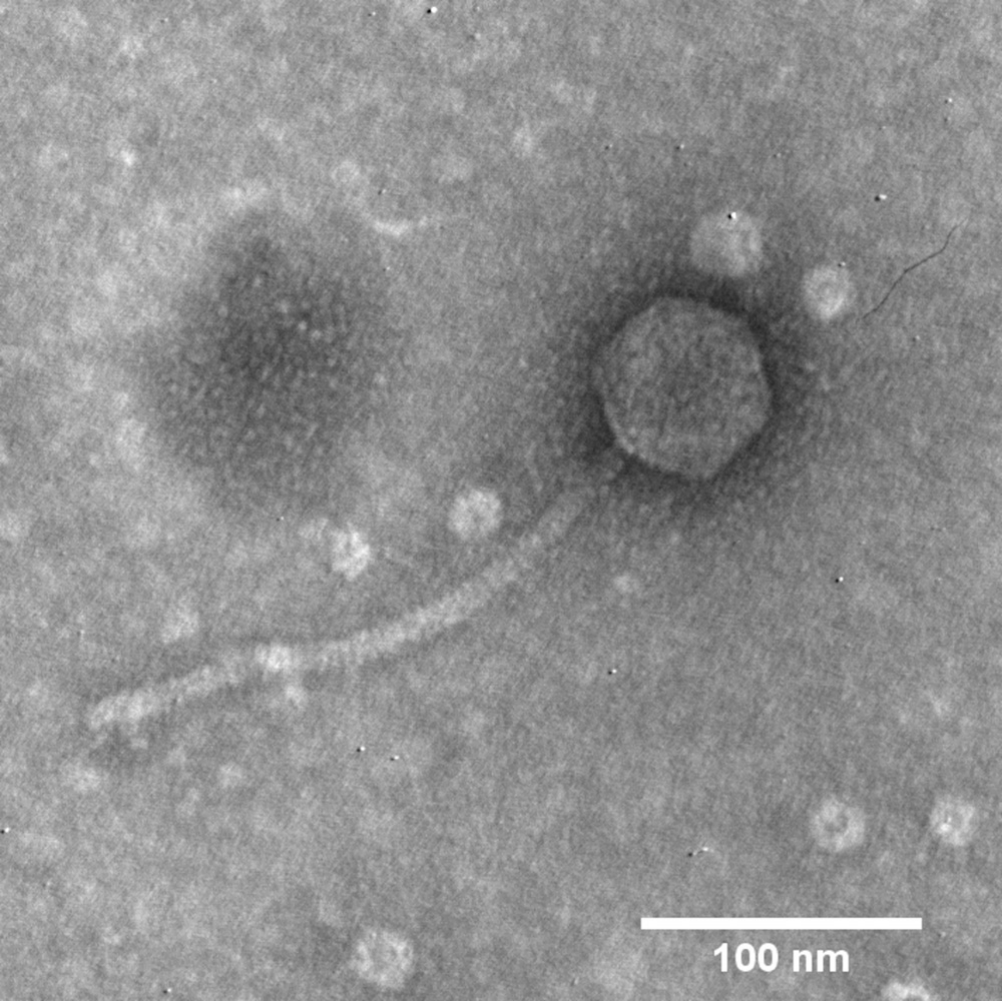
Transmission electron micrograph (TEM) of phage Suzuki. Phage particles were diluted with TEM buffer (20 mM NaCl, 10 mM Tris-HCl, pH 7.5, and 2 mM MgSO_4_) and captured on a freshly glow-discharged, Formvar carbon-coated grid. The grids were stained with 2% (wt/vol) uranyl acetate and observed on a JEOL 1200 EX TEM at 100 kV accelerating voltage at the Microscopy and Imaging Center at Texas A&M University.

Phage Suzuki was determined to be a siphophage (Fig. 1). The genome of Suzuki is 56,042 bp long with 62.6% G+C content and a 94.6% coding density. Out of 83 predicted protein-coding genes, 30 genes were assigned a putative function; among them is the predicted endolysin *N*-acetylmuramidase (InterPro accession no. IPR024408) needed for host lysis as part of a lysis cassette with nonembedded i- and o-spanins and a holin-antiholin pair. BLASTp comparisons (E value of <0.001) showed Suzuki shares 67 similar unique proteins with Xylella fastidiosa phage Sano (GenBank accession no. NC_042344), a 56-kb virulent siphophage with the potential to treat plant diseases caused by X. fastidiosa and *Xanthomonas* (21). At the whole-genome nucleotide level, Suzuki is most similar to Xylella fastidiosa phages Sano (78.13% overall similarity) and Salvo (GenBank accession no. NC_042345; 60.31% overall similarity) as determined by progressiveMauve.

### Data availability.

The genome of Suzuki was deposited in GenBank with accession number MZ326855. The associated BioProject, SRA, and BioSample accession numbers are PRJNA222858, SRR14095247, and SAMN18509290, respectively.
